# Skin side effects of chlorine solutions used for hand hygiene: a systematic review

**DOI:** 10.1186/2047-2994-4-S1-P9

**Published:** 2015-06-16

**Authors:** Z Kubilay, J Hopman, T Allen, H Edrees, B Allegranzi

**Affiliations:** 1 SDS, World Health Organization, Geneva, Switzerland; 2 Library, World Health Organization, Geneva, Switzerland

## Introduction

Chlorine solutions (CS), mostly containing sodium hypochlorite (SH), have been widely used for hand hygiene (HH) in the West African countries affected by the Ebola outbreak due to unavailability of alcohol-based handrub solutions and soap, easiness of use and “fear” factors leading to (false) sense of safety given by using an easily available disinfectant. However, no HH guidelines recommend the use of CS and concerns have been raised about skin tolerability among users.

## Objectives

We conducted a systematic review to investigate whether the use of CS causes skin side effects when used for HH.

## Methods

PubMed and EMBASE were searched on 26/09/14 with no time, age, human, language or geographical restrictions. *Contact Dermatitis* journal and the reference lists of relevant articles were also screened separately.

## Results

Out of 3241 hits, 14 articles about skin side effects were included; 10 case reports, 3 surveys and one comparative study. Only one case report was related to the use of SH 4-6% for HH by a veterinary surgeon who developed allergic contact dermatitis (CD) with a positive patch test to SH at dilutions 100 times lower than 4-6 %. In 5 case reports, CD with a variable severity was reported following use of SH for disinfection (2 papers) and for domestic cleaning (3papers); these cases had positive patch tests for SH; conversely, their experimental control groups showed either no reaction or very low-intensity skin reactions. Two papers reported severe dermatitis related to environmental cleaning with SH, and 2 others reported unusual systemic allergic reactions with accidental exposure and bathing the foot. One comparative study showed that SH was the most irritating product even at low concentrations when compared to alcohol, chlorhexidine and iodine. Finally, one survey among 706 nurses showed that 33.5% of cases of allergic or irritant CD had a history of chlorine exposure; 2 other surveys among cleaners showed higher prevalence of hand dermatitis with SH exposure.

## Conclusion

Overall, only one report described allergic CD related to use of high-concentration CS for HH. Very low-quality evidence shows that SH used for other purposes might cause skin irritations even at low concentrations following bare skin exposure.

## Disclosure of interest

None declared.

